# The Quality of Instructional YouTube Videos for the Administration of Intranasal Spray: Observational Study

**DOI:** 10.2196/23668

**Published:** 2020-12-30

**Authors:** Marije M Peters-Geven, Corine Rollema, Esther I Metting, Eric N van Roon, Tjalling W de Vries

**Affiliations:** 1 Department of Neonatology Isala Zwolle Netherlands; 2 Department of Clinical Pharmacy and Pharmacology Medical Centre Leeuwarden Leeuwarden Netherlands; 3 Faculty of Economics and Business University of Groningen Groningen Netherlands; 4 Groningen Research Institute of Pharmacy, Department of PharmacoTherapy, -Epidemiology and -Economy University of Groningen Groningen Netherlands; 5 Department of Paediatrics Medical Centre Leeuwarden Leeuwarden Netherlands

**Keywords:** allergic rhinitis, administration, intranasal spray, instruction videos, nasal, YouTube, educational video, corticosteroid, allergic, intranasal, allergy

## Abstract

**Background:**

Allergic rhinitis is a common disorder affecting both children and adults. Recommended treatment consists of intranasal corticosteroid spray administration, but only few patients administer the nasal spray in the correct technical manner. A wrong administration technique may result in side effects and affect the efficacy and adherence, thus making accurate administration instructions indispensable. Unfortunately, information about intranasal drug administration is generally not explained accurately, thereby leading to confusion among patients and inaccuracy in the self-administration of drugs.

**Objective:**

In this study, we analyzed instructional videos available on YouTube for the administration of nasal sprays for allergic rhinitis. Our aim was to determine if the videos provided instructions in accordance with the standardized nationwide patient protocol in the Netherlands for intranasal spray administration.

**Methods:**

Instructional videos for the administration of aqueous formulations of nasal spray for allergic rhinitis were found on YouTube. All videos were reviewed by 2 researchers and scored using the instructions from the Dutch standardized protocol. Correct instructions were given a score of 1, while incorrect or missing instructions were given a score of 0. The interrater reliability using Cohen ĸ was used to determine the differences in the scores between the researchers.

**Results:**

We identified 33 YouTube videos made by different health care professionals and pharmaceutical companies around the world. None of the videos displayed all the steps correctly, while 5 of the 33 (15%) videos displayed over 75% of the steps correctly. The median score of the correctly displayed steps was 11 out of 19 (range 2-17, IQR 6). The interrater reliability using Cohen ĸ was statistically significant (range 0.872-1.00, *P*<.001)*.* The steps “neutral position of the head,” “breathing out through the mouth,” and “periodically cleaning with water” scored the lowest and were incorrectly displayed in 28 (85%), 28 (85%), and 30 (91%) of the 33 videos, respectively.

**Conclusions:**

The findings of our study revealed that only few instructional videos on YouTube provided correct instructions for the administration of nasal sprays to patients. The inaccuracy of the instructions for nasal spray administration in the majority of the videos may lead to confusion in patients and incorrect use of nasal sprays. In the future, it is important to make evidence-based instructional videos that show patients the correct technique of nasal spray administration.

**Trial Registration:**

Not applicable

## Introduction

Allergic rhinitis is a common disorder affecting both children and adults [1,2]. The worldwide prevalence ranges from 5% to 32%, depending on age and geographics [2,3]. Although not life-threatening, this disorder has a major impact on patients’ daily activities and quality of life [4-6]. When the symptoms are persistent, the recommended treatment for allergic rhinitis consists of administration of intranasal corticosteroid sprays [7-9]. It is important to administer the nasal spray in the correct technical manner. A recent study has shown that only 6% of the patients used the correct administration technique of intranasal corticosteroid sprays as described in the patient information leaflet [10]. However, administering the spray in the correct manner is essential because it appears to influence the side effects, efficacy, and adherence [11]. Patients may receive instructions regarding correct administration from their health care professional or the patient information leaflet or they can find information on the internet. However, it is suspected that patients do not receive this information correctly. Indeed, a recent study has shown that Dutch health care workers do not provide their patients with correct administration instructions. In addition, instructions in patient information leaflets for the administration of intranasal corticosteroid sprays are incomplete and nonuniform in both the Netherlands and the United Kingdom [12]. Because of the lack of easily accessible information on this topic, a standardized nationwide patient protocol for intranasal corticosteroid spray administration was introduced in the Netherlands in 2019 [13]. This guideline has been drawn up and checked by various experts and is therefore peer-reviewed. Because this is the only peer-reviewed protocol available, it is considered as standard in the Netherlands. Since 2019, this protocol has been used by health care providers in primary and outpatient care. For patients, an illustrated instruction chart has been published based on this protocol. Besides the protocol and the patient information leaflets, different providers have posted web-based instructional videos for nasal spray administration to inform patients and health care providers. In this observational study, we investigated which instructions for the administration of nasal sprays are given in the videos that can be found on YouTube, and we compared these instructions with the Dutch protocol to see if the given instructions are correct.

## Methods

### Recruitment

Instructional videos on YouTube were found by using the keywords “How to use nasal spray,” “How do you use nasal spray,” “Usage nasal spray,” “Nasal spray instruction,” “Nasal spray technique,” “How to use nasal corticosteroids,” “How do you use nasal steroid spray,” “How do you use nasal steroids,” and “How do you use nasal corticosteroid sprays.” Brand names were included as keywords in an extensive search strategy. It turned out that these videos were already included, because publishers of these videos used the keyword “nasal spray” in their videos. Further, the same keywords were used on Google, but all pages were redirected to the already included YouTube videos.

All videos with English instructions for the nasal administration of an aqueous formulation with saline, antihistamines, and corticosteroids in a normal spray pump device were included. These sprays are all comparable with the spray pump devices that are available for patients in the Netherlands and need to be administered in the same way. Videos with other devices for nasal drug administration (such as nasal drops, nasal lavage, and spray pump devices with different user instructions compared to the normal spray pump device) and videos with nasal sprays for other indications (eg, naloxone) were excluded. Videos that provided only textual instructions were also excluded. No distinction was made between the creators of the instructional videos. All videos were collected and saved on the same day (October 24, 2019). The data collection flowchart is shown in Multimedia Appendix 1.

### Statistical Analysis

#### Scoring

All instructions in the videos were reviewed by 2 researchers (MMPG and CR) and scored using the instructions as described in the Dutch standardized protocol as mentioned before (Textbox 1). The protocol consists of 19 steps comprising the preparation, administration, and cleaning of the spray. Each step was scored. When a correct instruction was given, the score was 1; an incorrect or missing instruction was scored as 0. In the analysis, descriptive statistics were used to determine which instructions for the administration of nasal sprays are given in the YouTube videos.

**Assessed steps for administration of intranasal sprays based on the steps in the standardized Dutch protocol.**

**Steps for priming**

- Shake the spray.
- Remove the dust cap.
- Place thumb under the bottle and place index and middle fingers around the nozzle.
- Point the nozzle away.
- Squirt a few sprays in the air.

**Steps for daily use**

- Blow the nose.
- Shake the spray.
- Remove the dust cap.
- Place thumb under the bottle and place index and middle fingers around the nozzle.
- Keep the head straight.
- Close the other nostril.
- Point the end of the nozzle slightly outwards, away from the septum.
- Use contralateral hand position.
- Squirt a spray of mist while breathing in gently.
- Breathe out through the mouth.
- Repeat for the other nostril.
- Wipe the nozzle with a tissue.
- Replace the dust cap.
- Clean the nozzle once a week with warm water and let dry.

#### Assessment of Reliability

The interrater reliability using Cohen ĸ was used to determine the differences in the scores between the researchers. When differences in the scores were detected, the researchers reanalyzed the video and together determined the final score.

## Results

### Recruitment

A total of 33 videos were found and analyzed (details of the videos can be found in Table 1 and an overview of the included videos is shown in Multimedia Appendix 2). Most of the videos were made by physicians (20/33, 61%) from the United States (19/33, 58%) and were created for adults (30/33, 91%) by using intranasal corticosteroids (17/33, 52%).

**Table 1:** Characteristics of the analyzed YouTube videos (N=33).

Characteristics		n (%)
**Professionals who created the videos**		
	Pharmacist	3 (9)
	Physician	20 (61)
	Pharmaceutical company	6 (18)
	Other/unknown	4 (12)
**Instruction for children**		
	Yes	3 (9)
	No	30 (91)
**Date of publication on YouTube**		
	<6 months before analysis	2 (6)
	6-12 months before analysis	0 (0)
	>12 months before analysis	31 (94)
**Medication type**		
	General	15 (46)
	Corticosteroids	17 (52)
	Antihistamines	1 (3)
**Video type**		
	Recorded with image and sound	29 (88)
	Animation with spoken instructions	4 (12)
**Origin**		
	United Kingdom	3 (9)
	United States	19 (58)
	Australia	4 (12)
	India	2 (6)
	Canada	1 (3)
	Other/unknown	4 (12)

### Statistical Analysis

#### Scoring

There was no video that showed all the steps correctly. The 3 videos with the highest score displayed 17 of the 19 steps correctly. Only 5 out of 33 (15%) videos displayed over 75% of the steps correctly. The median of the correctly displayed steps was 11, with a range between 2 and 17 and an interquartile range of 6. Instructions regarding removing the dust cap, the correct hand position, and repeating for the other nostril were correctly displayed most of the time, that is, in 32 (97%), 29 (88%), and 27 (82%) of the 33 videos, respectively. Keeping the head straight, breathing out through the mouth after spraying, and periodically cleaning the nozzle with water scored the lowest. These steps were incorrectly displayed in 28 (85%), 28 (85%), and 30 (91%) of the 33 videos, respectively. The results for each step are shown in Table 2.

#### Assessment of Reliability

There was a high degree of agreement in the scores between the 2 researchers, and the majority of the Cohen ĸ values were equal to 1 (lowest Cohen ĸ=0.872, Table 2).

**Table 2:** Number of steps carried out correctly and incorrectly in the analyzed videos, based on the steps in the standardized Dutch protocol (N=33).

Steps in instructional videos		Instruction carried out (Researcher 1), n (%)	Instruction carried out (Researcher 2), n (%)	Interrater reliability, Cohen ĸ	*P* value	Instruction carried out (conclusion), n (%)	Instruction not carried out (conclusion), n (%)
**Steps for priming**							
	Shake the spray	17 (52)	17 (52)	1.00	<.001	17 (52)	16 (48)
	Remove the dust cap	23 (70)	23 (70)	1.00	<.001	23 (70)	10 (30)
	Place thumb under the bottle and place index and middle fingers around the nozzle	21 (64)	21 (64)	1.00	<.001	21 (64)	12 (36)
	Point the nozzle away	21 (64)	21 (64)	1.00	<.001	21 (64)	12 (36)
	Squirt a few sprays in the air	22 (67)	22 (67)	1.00	<.001	22 (67)	11 (33)
**Steps for daily use**							
	Blow the nose	17 (52)	17 (52)	1.00	<.001	17 (52)	16 (48)
	Shake the spray	22 (67)	22 (67)	1.00	<.001	22 (67)	11 (33)
	Remove the dust cap	32 (97)	32 (97)	1.00	<.001	32 (97)	1 (3)
	Place thumb under the bottle and place index and middle fingers around the nozzle	28 (85)	29 (88)	0.872	<.001	29 (88)	4 (12)
	Keep the head straight	5 (15)	5 (15)	1.00	<.001	5 (15)	28 (85)
	Close the other nostril	17 (52)	17 (52)	1.00	<.001	17 (52)	16 (48)
	Point the end of the nozzle slightly outwards, away from the septum	21 (64)	22 (67)	0.933	<.001	21 (64)	12 (36)
	Use contralateral hand position	13 (39)	13 (39)	1.00	<.001	13 (39)	20 (61)
	Squirt a spray of mist while breathing in gently	19 (58)	21 (64)	0.874	<.001	20 (61)	13 (39)
	Breathe out through the mouth	5 (15)	5 (15)	1.00	<.001	5 (15)	28 (85)
	Repeat for the other nostril	27 (82)	27 (82)	1.00	<.001	27 (82)	6 (18)
	Wipe the nozzle with a tissue	9 (27)	9 (27)	1.00	<.001	9 (27)	24 (73)
	Replace the dust cap	17 (52)	17 (52)	1.00	<.001	17 (52)	16 (48)
	Clean the nozzle once a week with warm water and let dry	3 (9)	3 (9)	1.00	<.001	3 (9)	30 (91)

## Discussion

### Principal Results

We found that none of the surveyed videos provide patients with correct instructions for the administration of nasal sprays as described in the standardized Dutch protocol. The instructors in the video either did not use a technique that is in line with the steps in the protocol or they showed only a few steps of the administration technique correctly. Instructions regarding keeping the head straight, breathing out through the mouth after spraying, and periodically cleaning the nozzle with water were incorrectly displayed in most of the videos. In the majority of the videos, the instructors recommended to bend the head forward during administration, instead of keeping the head straight. Exhalation through the mouth seemed to be not a conscious step specified by the instructors and was therefore not observed in the majority of the videos. Cleaning the nozzle is often not mentioned by the instructors and this indicates that the importance of this step is unknown.

The best administration technique of nasal sprays in relation to optimal treatment outcomes is marginally substantiated with research data. This may result in variations in the instructions used for the administration of nasal sprays, which is also what we observed in the web-based instructional videos. In the literature, there are differences in the instructions regarding the optimal head position, angle of the spray in the nose, and the way patients need to breathe in while spraying. However, there are studies that have clarified this. Benninger et al [14] have shown that when using intranasal corticosteroid sprays, there is no difference in the distribution of the spray for different positions of the head. Therefore, their advice is to keep the head in a neutral position. They also advise aiming the point of the nozzle outwards and away from the septum to avoid side effects such as epistaxis. Ganesh et al [11] have found that using a contralateral hand technique (using the left hand for the right nostril and vice versa) not only ensures a better effect of the spray and causes fewer side effects compared to ipsilateral administration but is also accompanied by improved compliance. The correct way to breathe in was explained by Tay et al [15]. They found that a gentle inspiration technique improves the intranasal distribution of the medication. The standardized protocol in the Netherlands for the administration of intranasal corticosteroid sprays is based on these findings and was developed to overcome the variation in the instructions [13]. We have assumed that the delivery techniques of all other sprays, in addition to intranasal corticosteroid sprays, are identical; for this reason, the Dutch protocol was used as the standard.

Previous research has shown that the majority of Dutch health care workers do not know how to administer intranasal corticosteroid sprays correctly; this may prevent them from being able to give adequate instructions to their patients [16]. Moreover, information in Dutch and British patient information leaflets is incomplete and nonuniform [12]. An increasing number of Europeans are looking for information on the internet. In the past 10 years, the percentage of people seeking health-related information on the internet has increased from 32% in 2009 to 53% in 2019 [17]. Benetoli et al [18] investigated the web-based behavior of people searching for health-related information on the internet and found that YouTube was broadly assessed for learning about medical procedures. It is therefore plausible that patients with allergic rhinitis might use YouTube for information about the correct administration of their nasal sprays. However, less is known about how these YouTube videos, easily reaching a broad audience, may affect patients [19]. This study shows that the video instructions for the administration of nasal sprays available on YouTube are of low quality, which can have important implications. It is therefore essential that correct information becomes easily available for patients with allergic rhinitis—incorrect information leads to incorrect use, and it may be confusing for patients when there are differences among the instructions they receive. As a result, patients will not recognize the need for correct use and will pay less attention when correct instructions are given. For asthma, multiple studies have shown that proper instructions ensure a better effect of the medication and that instructions must be repeated at least twice for good effect [20,21]. This indicates that there is a shortage of good research on this topic regarding nasal sprays.

We realize that everyone can make an instructional video and post it on YouTube, which means that incorrect information can be widely available. Yet, for most of the analyzed videos, the information was provided by a health care provider, which increases the trustworthiness of the video. This implies that there is a lack of substantiated knowledge about the correct administration technique of nasal sprays and that health care providers are providing instructions based on their own insights. Health care providers should be aware of the importance of the correct administration technique for nasal sprays and provide proper instructions both on the internet and in the consulting room. More attention needs to be drawn to the Dutch protocol, which can facilitate more uniform instructions given by health care professionals. In addition, health care providers should warn their patients about the low quality of web-based instructional videos, ask where patients receive their information, and explain to the patients why correct administration is so important.

### Comparison With Prior Work

As far as we know, this is the first study that evaluates the instructions available in YouTube videos for the administration of nasal sprays. However, there are a few studies that are similar to ours. It is known that the administration techniques and instructions for the treatment of lung diseases such as asthma and chronic obstructive pulmonary disease are of great importance for symptom control [20,21]. Eudaley et al [22] investigated the quality of the instructions in videos on YouTube for administration of the RESPIMAT SOFT MIST inhaler. They found that none of the videos available on YouTube included all of the necessary instruction steps for correct administration, and in only 4 out of 35 videos (11%), all the steps for daily use were included correctly. This finding corresponds to our findings. For rheumatological diseases also, instructional videos for the administration of treatment are available on YouTube. Two studies about the quality of the instructions in these videos concluded that most of the information is misleading and potentially dangerous for patients [23,24]. This underscores our findings and emphasizes the need and importance of good instructional videos on YouTube.

### Strengths and Limitations of Our Study

One strength of this study is that videos were found in a way that patients might also look for them; therefore, it reflects daily practice. Another strength of this study is that the videos were analyzed by 2 researchers, with a high degree of agreement in the scores (Cohen ĸ was used). However, this study also has some limitations. First, all videos were collected on the same day, but what is offered on YouTube changes continuously. It is possible that not all currently available videos are included in the study, although we used broad search terms to find all relevant videos. Second, this study evaluated differences between a standard protocol for nasal spray administration and protocols shown in web-based instructional videos. However, it is unclear whether patients actually look for this information as a guide to learning the correct administration technique, thereby affecting efficacy. Besides, the administration instructions in the videos are compared here with the instructions in the Dutch intranasal corticosteroid spray administration protocol but it has not yet been established whether these instructions are identical for all nasal sprays. For this reason, future research on the relation between administration technique and the efficacy of all available nasal sprays would add value. Translating the Dutch protocol into other languages and into a video for patients is also important. In addition, it is important to continuously evaluate the quality of information available on YouTube and to find ways to guarantee good quality of the available content.

### Conclusions

This study shows that the majority of the instructional videos that can easily be found on YouTube do not provide patients the correct instructions for the administration of nasal sprays. This can lead to confusion in patients and to incorrect use of the nasal spray. In the future, the Dutch protocol should be translated into other languages, and evidence-based instructional videos should be made, which show patients the correct administration technique.

## Appendix

Multimedia Appendix 1 Data collection flowchart.

Multimedia Appendix 2 Overview of the YouTube videos.
