# Supplementary figures and images for: The Quality of Instructional YouTube Videos for the Administration of Intranasal Spray: Observational Study
Source: JMIR Med Educ. 2020 Dec 30;6(2):e23668. doi: 10.2196/23668 (PMC7806442; doi:10.2196/23668)

Appendix 1: Data collection flow chart


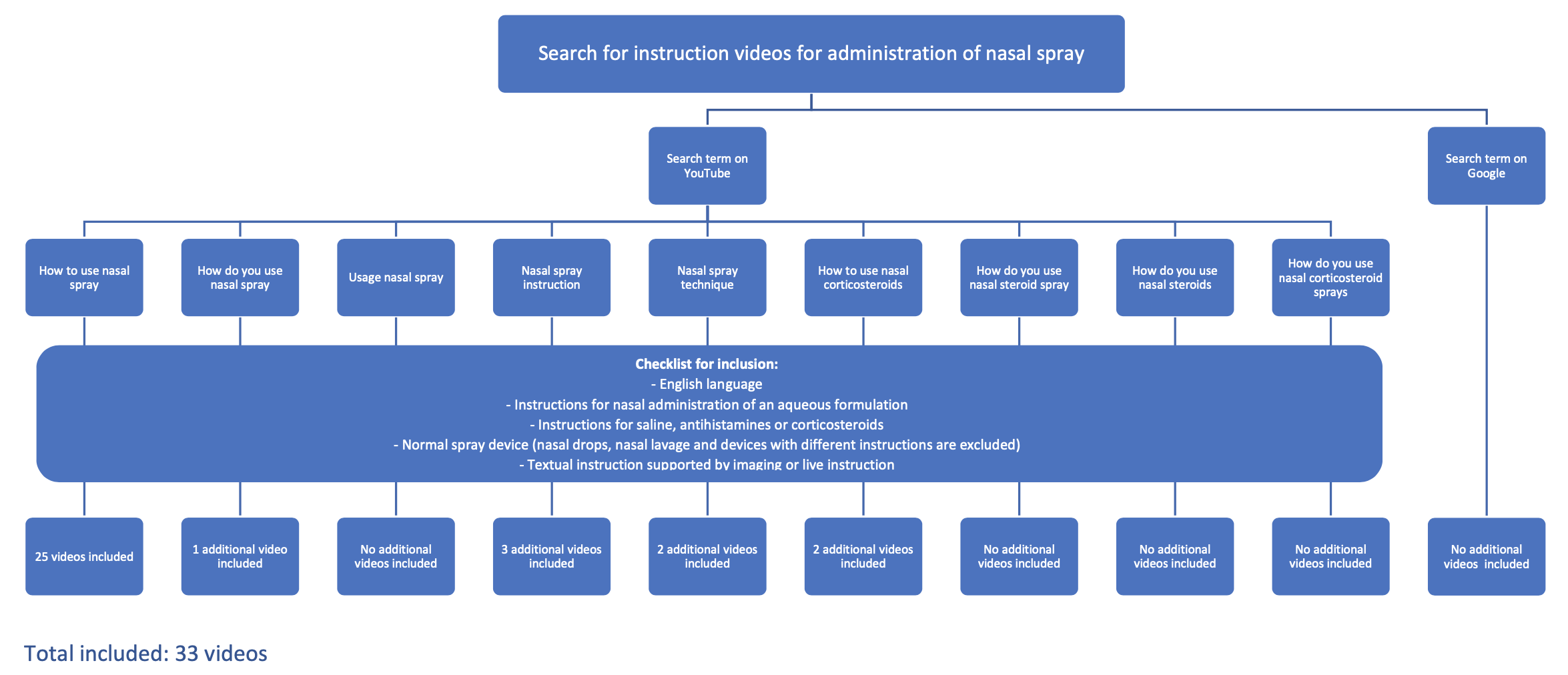

Supplement: Multimedia Appendix 1 [file mededu_v6i2e23668_app1.docx]
