# Supplementary material for: The Quality of Instructional YouTube Videos for the Administration of Intranasal Spray: Observational Study
Source: JMIR Med Educ. 2020 Dec 30;6(2):e23668. doi: 10.2196/23668 (PMC7806442; doi:10.2196/23668)
Supplement: Multimedia Appendix 2 [file mededu_v6i2e23668_app2.docx]

## **Appendix 2: Overview of the YouTube videos**

*This list shows the analysed YouTube videos and shown are the name of the video, the publisher? of the video, the date of publication, the duration of the video and the URL. Videos were searched by six different keywords and are listed below per keyword (last availability check at October 21, 2020).*

## 1. Results for: ‘How to use nasal spray’

1. How To Use Nasal Spray | How To Use Nasal Spray Properly | Nasal Spray Technique (2018). Abraham the Pharmacist. May 2018. Duration 4.29 min.
   <https://www.youtube.com/watch?v=mYQhIaoIKj0>
2. Mayo Clinic Minute: Combat allergies like a pro by learning how to use your nasal spray properly. Mayo Clinic. April 2018. Duration 1.00 min.
   <https://www.youtube.com/watch?v=ICqBAN28Tgo>
3. How to Use Nasal Sprays Properly. Apotex Inc. March 2015. Duration 2.03 min.
   <https://www.youtube.com/watch?v=r4rWN8T89yU>
4. Helping Your Child Use Nasal Spray. Ann & Robert H. Lurie Children’s Hospital of Chicago. September 2014. Duration 2.59 min
   <https://www.youtube.com/watch?v=8Qi1S1nR3PY>
5. Dr. Gregory Abbas: The Proper Use of Nasal Spray HD. Advanced ENT & Allergy. January 2014. Duration 3.08 min.
   <https://www.youtube.com/watch?v=tnEre7FHUAQ>
6. Choosing nasal sprays for your child. Nationwide Childrens. August 2013. Duration 1.25 min.
   Download available for request, online not longer available.
7. How to use a steroid nasal spray. Clancy ENT. October 2016. Duration 1.50 min.
   <https://www.youtube.com/watch?v=s6umh_1Vigs>
8. How to use a Nasonex nasal inhaler spray. Impagination. October 2012. Duration 3.42 min.
   <https://www.youtube.com/watch?v=f1Xh3ABQaI8>
9. How to Use Nasal Spray - Nasal Spray Technique for Allergy and Sinus - Dr. Joorabchi. Doctor Sina. June 2019. Duration 2.40 min.
   <https://www.youtube.com/watch?v=UTGLV9mh1Ic>
10. How To Use Nasal Spray (English Language). Allergy Center Islamabad. February 2018. Duration 2.13 min.
    <https://www.youtube.com/watch?v=h42SyhNWy64>
11. Control allergic rhinitis – use nasal spray. Breathefreetv. February 2015. Duration 2.53 min.
    <https://www.youtube.com/watch?v=nS04HjuOCUA>
12. How to use nasal spray. AshevilleVideo. February 2012. Duration 1.19 min.
    <https://www.youtube.com/watch?v=iaZdNhp8_40>
13. Flonase Allergy Relief How to use. Flonase US. February 2015. Duration 1.28 min.
    <https://www.youtube.com/watch?v=85I7dtLIWWU>
14. How To Use Nasal Spray Properly | Nasal Spray Technique 2019 | How To Use Nasal Spray. Dr. Amrapali Pawar. August 2019. Duration 2.32 min.
    <https://www.youtube.com/watch?v=vWztCR8JlH0>
15. How to use a prescription nasal spray. Majd MD. July 2018. Duration 2.34 min.
    <https://www.youtube.com/watch?v=iR0e1Qv5jWA>
16. How To Use Your Nasal Steroid Spray | Dr. Thomas Lamperti | Seattle Facial Plastic Surgeon. DrLamperti. January 2011. Duration 2.40 min.
    <https://www.youtube.com/watch?v=_r89dNRMdYs&t=3s>
17. RHINOCORT® Allergy Spray: How To Use | RHINCORT®. Rhinocort®. July 2016. Duration 0.51 min.
    <https://www.youtube.com/watch?v=O0suJ5ALe6g>
18. How to use a Rhinocort nasal inhaler spray. Impagination. October 2012. Duration 3.22 min.
    <https://www.youtube.com/watch?v=wQ1V7y0LfII>
19. Nasal spray technique. Gary Steven. June 2013. Duration 2.50 min. <https://www.youtube.com/watch?v=kHeYBUltC34>
20. How to use nasal spray. Onlineallergist. August 2009. Duration 0.39 min
    <https://www.youtube.com/watch?v=wQbyC5cpEQI>
21. How to use a nasal spray for hay fever and allergies. Asthma UK and British Lung Foundation Partnership. December 2016. Duration 1.29 min.
    <https://www.youtube.com/watch?v=8tWuleHL7Pk>
22. Proper use of nasal spray. Midwest ENT. August 2014. Duration 2.45 min.
    <https://www.youtube.com/watch?v=kiEOIDljjow>
23. Spiroflut Nasal spray. Aviactive Creatives. September 2018. Duration 2.53 min.
    <https://www.youtube.com/watch?v=x-9HN-6ieb0>
24. Bayer Nasonex Allergy How to Spray Video Tutorial 2017. Nasonex Allergy Australia. August 2017. Duration 2.00 min.
    <https://www.youtube.com/watch?v=aYHmllrJBlY>
25. Nasal spray use: correct technique. Family Allergy, Asthma, Immunology & Sinus Center P.C.. July 2014. Duration 1.45 min.
    <https://www.youtube.com/watch?v=uMtAUs6MUaM>

## 2. Additional results for ‘How do you use nasal spray’

1. Medicine Management - How to Administer Nasal Sprays. Medicine Management. September 2014. Duration 1.33 min.
   <https://www.youtube.com/watch?v=R178eS66nD8>

## 3. Additional results for ‘usage nasal spray’

No additional results.

## 4. Additional results for ‘Nasal spray instruction’

1. Dr. Adappa and Dr. Palmer Teach You How to Use Nasal Spray. Rhinology at Penn. May 2016. Duration 2.30 min.
   <https://www.youtube.com/watch?v=6mlUbV6m-Rs>
2. How to use nasal steroids. TexasSinusCenter. April 2015. Duration 1.29 min.
   <https://www.youtube.com/watch?v=KzYQD8Cpbbw>
3. How to use a nasal spray. Medvize. May 2017. Duration 2.39 min.
   <https://www.youtube.com/watch?v=8BpyZARQ8cE>

## 5. Additional results for ‘Nasal spray technique’

1. How to use a nasal spray. InsideChild.org. October 2018. Duration 2.12 min.
   <https://www.youtube.com/watch?v=45dOz18xqPE>
2. Improve Your Response to Pump-Based Nasal Sprays. James Thompson. January 2013. Duration 2.33 min.
   <https://www.youtube.com/watch?v=xjzrSD87DsY>

## 6. Additional results for ‘how to use nasal corticosteroids’

1. How to Use a Nasal Steroid with Emory's Dr Shams. Emory University. March 2017. Duration 1.31 min.
   <https://www.youtube.com/watch?v=T6VsZisQr7s>
2. Administration of a Nasal Corticosteroid with Dr. Anjali Bhaisin. Dr. Anjali Bhaisin. March 2017. Duration 0.47 min.
   <https://www.youtube.com/watch?v=dh1S-HVF1fA>
